# The Keap1-Nrf2 System: A Mediator between Oxidative Stress and Aging

**DOI:** 10.1155/2021/6635460

**Published:** 2021-04-19

**Authors:** Chao Yu, Jian-Hui Xiao

**Affiliations:** ^1^Zunyi Municipal Key Laboratory of Medicinal Biotechnology, Affiliated Hospital of Zunyi Medical University, 149 Dalian Road, Huichuan District, Zunyi 563003, China; ^2^Guizhou Provincial Research Center for Translational Medicine, Affiliated Hospital of Zunyi Medical University, 149 Dalian Road, Huichuan District, Zunyi 563003, China

## Abstract

Oxidative stress, a term that describes the imbalance between oxidants and antioxidants, leads to the disruption of redox signals and causes molecular damage. Increased oxidative stress from diverse sources has been implicated in most senescence-related diseases and in aging itself. The Kelch-like ECH-associated protein 1- (Keap1-) nuclear factor-erythroid 2-related factor 2 (Nrf2) system can be used to monitor oxidative stress; Keap1-Nrf2 is closely associated with aging and controls the transcription of multiple antioxidant enzymes. Simultaneously, Keap1-Nrf2 signaling is also modulated by a more complex regulatory network, including phosphoinositide 3-kinase (PI3K)/protein kinase B (Akt), protein kinase C, and mitogen-activated protein kinase. This review presents more information on aging-related molecular mechanisms involving Keap1-Nrf2. Furthermore, we highlight several major signals involved in Nrf2 unbinding from Keap1, including cysteine modification of Keap1 and phosphorylation of Nrf2, PI3K/Akt/glycogen synthase kinase 3*β*, sequestosome 1, *Bach1*, and *c*-*Myc*. Additionally, we discuss the direct interaction between Keap1-Nrf2 and the mammalian target of rapamycin pathway. In summary, we focus on recent progress in research on the Keap1-Nrf2 system involving oxidative stress and aging, providing an empirical basis for the development of antiaging drugs.

## 1. Introduction

Aging is a fundamental and complex physiological process that compromises health, causing multiple chronic diseases. Aging and antiaging are universal concerns in the life sciences. Over the past few decades, researchers have continuously explored the underlying mechanisms of aging and antiaging interventions. Among the theories proposed to explain aging, damage accumulation driven by oxidative stress is one of the most accepted ones [1].

The concept of oxidative stress can be traced back to the 1950s and has been studied since the 1970s [2]. The term “oxidative stress,” popularized by Sies and Cadenas [3], was defined as “a disturbance in the prooxidant-antioxidant balance in favor of the former, leading to potential damage,” and later, Jones [4] introduced a new definition of oxidative stress as “a disruption of redox signaling and control.” It involves the excessive generation of reactive oxygen species (ROS) and reactive nitrogen species (RNS), such as peroxynitrite (ONOO^−^), hydrogen peroxide (H_2_O_2_), nitric oxide (NO), hydroxyl radicals (HO^·^), and superoxide anion radicals (O_2_^·-^) [4]. Sources of ROS and RNS in organisms are abundant under physiological and pathological conditions. Cells contain multiple sources for ROS, including the mitochondria, endoplasmic reticulum, peroxisomes, NAD(P)H oxidases, and monoamine oxidases [5, 6]. Of these, complexes I and III, involved in mitochondrial oxidative phosphorylation, are the largest contributors to cellular ROS production [7, 8]. High cellular ROS levels result from an imbalance between ROS production and the antioxidant system. This increase in ROS damages DNA, cell membranes, and proteins, leading to the development of aging-related and neurodegenerative diseases [9, 10]. Recent studies have corroborated the idea that DNA damage has a significant effect on aging [11, 12]. Over the past two decades, we have rapidly increased our understanding of the role of DNA damage response in cancer and aging [13]. Research in a wide variety of animal models from *Caenorhabditis elegans* to mammals has shown that cellular autonomy and systematic DNA damage response mechanisms coordinate adaptive responses, thereby enhancing maintenance in aging organisms and accumulating DNA damage more gradually [14, 15]. Interestingly, recent evidence has shown that routine exercise attenuates aging-induced oxidative stress [16]. Similarly, constraint-induced movement in mice effectively inhibits aging, whereas aging is accelerated with greater cellular oxidative stress [17]. Thus, an increasing body of evidence suggests that accumulating ROS, dysfunctional mitochondria, and DNA damage play an important role in the aging process.

To deal with oxidative damage, the body is equipped with an efficient defense system that detoxifies and eliminates harmful chemicals and inactivates ROS. Nuclear factor-erythroid 2-related factor 2 (Nrf2) is a master regulator of multiple antioxidant enzymes; it modulates cell redox balance and senses the status of cellular oxidative stress. This is done by stimulating the activity of components of antioxidant defense, such as superoxide dismutase (SOD), glutathione peroxidase (GSH-Px), heme oxygenase-1 (HO-1), glutathione reductase, thioredoxin reductase, ferritin, and NAD(P)H:quinone oxidoreductase 1 (NQO1) [18, 19]. Therefore, Nrf2 is a key regulator in the signaling pathway for lifespan extension because of its role in regulating antioxidant expression [20]. Activation of the Nrf2-related antioxidant defense system prevents cell senescence, while inhibiting Nrf2 activity significantly promotes cell senescence [21], indicating that Nrf2 has a protective effect on aging. Furthermore, Nrf2 expression and activity decrease during aging [22, 23].

Kelch-like ECH-associated protein 1 (Keap1), a master negative regulator of Nrf2 discovered in 1999 [24], has a broad, complex, tram-track, bric-a-brac structure at its *N*-terminus. Keap1 is the site of action for cullin-dependent E3 ubiquitin ligase, which degrades Nrf2 [25, 26]. Most Nrf2 inducers, such as *tert*-butylhydroquinone (tBHQ) and itaconate [27, 28], are electrophilic and readily react with cysteine thiol groups in Keap1, activating the Keap1-Nrf2 signaling pathway and triggering a protective antioxidant response. Under oxidative stress, Keap1 undergoes a conformational change that causes Nrf2 to dissociate. The latter protein then aggregates into the nucleus and forms a heterodimer with musculoaponeurotic fibrosarcoma (Maf), which combines with antioxidant response elements (AREs) to initiate the transcription of multiple antioxidants [29].

The Keap1-Nrf2 system has been widely studied and is considered a key cellular defense mechanism. However, multiple factors regulate the Keap1-Nrf2 system, posing a formidable challenge to unraveling the underlying mechanism linking the Keap1-Nrf2 signaling pathway to aging. Researchers are currently working to identify candidate antioxidant-related agents that activate Nrf2 for development into antiaging drugs and for treating aging-related diseases. These efforts are largely dependent on elucidating the correlation between Keap1-Nrf2 signaling and oxidative stress. Therefore, in this review, we present a compilation of the regulation of Keap1-Nrf2 signaling to provide new ideas for the identification and development of antiaging agents.

## 2. Activation of Nrf2 and Aging

The role of Nrf2 in antioxidative stress is well defined [30]. Only within the last few years, however, evidence demonstrates that Nrf2 activity is repressed in aging. A recent study showed that Nrf2 deletion caused widespread aging-related transcriptomic changes in age-related diseases [31]. Nrf2 signaling was impaired in the retinal pigment epithelium in aging mice [32]. Furthermore, aging was associated with a loss of Nrf2 activity, particularly from regions that had high Nrf2 activity in young animals [33]. Interestingly, acute aerobic exercise activates Nrf2 on peripheral blood mononuclear cells *in vivo* in both the old and young adults, but the nuclear accumulation of Nrf2 was attenuated in older adults [34]. These findings suggest that Nrf2 signaling is closely associated with aging and age-related diseases. However, how does Nrf2 signaling influence aging and age-associated diseases?

As mentioned above, Nrf2 levels have been shown to decrease with aging, and there is a positive relationship between Nrf2 activity and species longevity. Therefore, the regulation and downstream pathways of Nrf2 have received special attention. Nrf2 regulates various downstream antioxidation and detoxification enzymes such as NQO1, HO-1, SOD, catalase (CAT), and glutamate-cysteine ligase catalytic subunit (GCLC) by binding to the antioxidant response element in their promoter regions [35]. For example, trehalose and chitosan oligosaccharide all showed an antiaging effect by promoting the nuclear translocation of Nrf2 and subsequently activating the expression of downstream target genes HO-1, NQO1, and CAT in aging mice [36, 37]. Furthermore, recent studies showed that the activation of multiple signaling pathways, such as phosphatidylinositol 3-kinase (PI3K)/protein kinase B (Akt), protein kinase C (PKC), and mitogen-activated protein kinase (MAPK), facilitates the release of Nrf2 from Keap1 and subsequent translocation for the induction of various antioxidant and detoxification enzyme expressions [19, 38, 39]. Intriguingly, Lewis et al. found that high levels of Nrf2 signaling activity in naturally long-lived naked mole rats are not due to increased expression of Nrf2 protein but rather are due to reduced expression of its negative regulators Keap1 and *β*-transducin repeat-containing protein (*β*-TrCP) [40]. Rapamycin also shows a similar mechanism to inhibit cell senescence and increase longevity [41]. In addition, Nrf2 activation or overexpression might not be enough to prolong healthy life in aging models. The constitutive activation or overexpression of Nrf2 even can cause deleterious outcomes. Rajasekaran et al. found that chronic activation of Nrf2 causes a hyperreductive state and leads to hypertrophic cardiomyopathy in the cardiac-specific transgenic mice [42]. Nrf2 overexpression increases the risk of high tumor mutation and induces drug resistance in cancer patients [43, 44].

**Figure 1:**
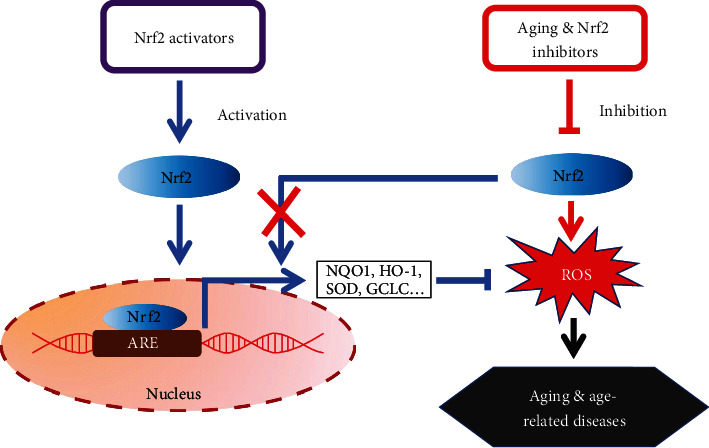
Simplistic schematic of the relationship between Nrf2 and aging.

Taken together, accumulating evidence suggests that the Nrf2 plays a key role in oxidative stress resistance and may be directly linked to the healthspan and lifespan of the organism (Figure 1). However, there are numerous targets on the Nrf2 signaling that may show positive effects on delaying aging. Thus, there is still a long way ahead to understand Nrf2 signaling during aging. Here, we provide a review of that area in order to provide the context for our discussion of how Nrf2 activation affects aging.

## 3. Mechanisms of Nrf2 Dissolution from Keap1

### 3.1. Modification of Keap1 Cysteine Residues

**Figure 2:**
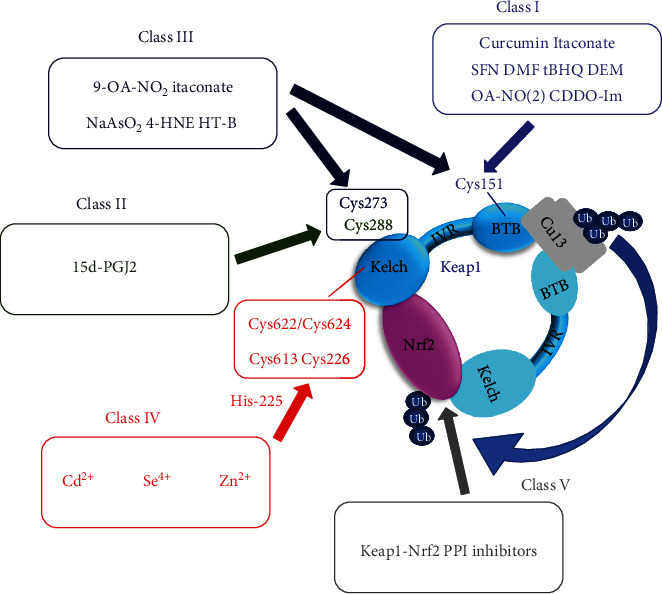
Different classes of activators of Keap1. Several representative compounds are shown and are divided into Classes I–V (further classified using the criteria established by Suzuki and Yamamoto [55] and based on current studies). 9-OA-NO_2_: 9-nitro-octadec-9-enoic acid; NaAsO_2_: sodium meta-arsenite; 4-HNE: 4-hydroxy-nonenal; SNAP: *S*-nitroso-N-acetylpenicillamine; 15d-PGJ2: 15-deoxy-prostaglandin J2; DMF: dimethylfumarate; CDDO-Im: 1-[2-cyano-3,12-dioxooleana-1,9(11)-dien-28-oyl]imidazole; tBHQ: *tert*-butylhydroquinone; DEM: diethylmaleate; SFN: sulforaphane; HT-B: hydroxytyrosol butyrate; PPI: protein-protein interaction.

Keap1, a ubiquitous Nrf2 regulator, is a major target in the discovery of antiaging drugs. Keap1 comprises two canonical domains: the *N*-terminal BTB and the *C*-terminal DC (or Kelch) domains, connected by an intervening region (IVR) [24]. Keap1 contains highly conserved, reactive cysteine residues that act as electrophilic sensors responding to endogenous and exogenous ROS. Studies have revealed three major half-cysteine residues of Keap1: Cys151 in the BTB domain and Cys273 and Cys288 in the IVR. These residues are critical for modulating the E3 ubiquitin ligase activity of the Keap1-Cul3 complex [45, 46]. Of the three half-cysteines, Cys151 is the main sensor of oxidative stress that disrupts the Keap1-Cul3 interaction and causes Nrf2 dissolution from Keap1 [47, 48]. In contrast, Cys273 and Cys288 play a crucial role in the Keap1 conformational change under oxidative modification [49]. Most importantly, while specific cysteine sensors have evolved in response to different inducers, there is redundancy between sensors, enabling continued oxidant accumulation even with a loss-of-function mutation in a given Keap1 sensor [28, 45, 46, 49]. This flexibility is extremely important and ensures cell survival by preventing excessive glutathione consumption and subsequent oxidative stress. Furthermore, Saito and colleagues have confirmed that Cys151, Cys273, and Cys288 function collaboratively in sensing 9-nitro-octadec-9-enoic acid, sodium meta-arsenite, and 4-hydroxy-nonenal [49]. Their findings also suggest that *S*-nitroso-*N*-acetylpenicillamine, 1-[2-cyano-3,12-dioxooleana-1,9(11)-dien-28-oyl]imidazole, tBHQ, diethylmaleate, sulforaphane, and dimethylfumarate are Cys151-preferring inducers [49]. Furthermore, other cysteine residues (e.g., Cys226, Cys613, and His225) are involved in the sensing function of Keap1 and are necessary for detecting Cd^2+^, As^3+^, Se^4+^, and Zn^2+^ [45, 50]. The H_2_O_2_ sensing ability was originally thought to be dependent on a conformational change in Keap1, induced through a disulfide bond forming between Cys226 and Cys613 [51]. However, a recent study has found that the conformational change occurs via the synergy of Cys226, Cys613, and Cys622/Cys624, inactivating Keap1 and stabilizing Nrf2 [52]. In addition, H_2_O_2_ mediates Keap1 phosphorylation at Ser^104^, Ser^53^, and Ser^293^ in response to oxidative stress [53, 54]. Given that different chemicals can target different cysteine sites in Keap1 to regulate Nrf2, chemical Nrf2 inducers have been divided into at least five categories as shown in Figure 2: I (Cys151 preferring), II (Cys288 preferring), III (Cys151/Cys273/Cys288 collaboration preferring), IV (Cys151/Cys273/Cys288 independent), and V (Keap1-Nrf2 protein-protein interaction) [55].

Inflammation is closely associated with aging [56]. Inflammation produces large amounts of ROS that induce oxidative damage in DNA, membrane lipids, and proteins, further contributing to aging [57]. Itaconate is a novel and potent Nrf2 activator that plays an anti-inflammatory role through alkylating Cys151, Cys257, Cys288, Cys273, and Cys297 in Keap1, enabling Nrf2 to increase the expression of downstream antioxidant and anti-inflammatory genes [27]. The itaconate derivative, 4-octyl itaconate, protects neuronal cells from H_2_O_2_ by targeting Keap1 [58]. The discovery of three important cysteine residues of Keap1 and several other half-cysteine sensors elucidates the oxidative stress-induced transcription of Nrf2-dependent genes, providing a reliable, theoretical basis for unveiling the aging mechanism and the development of antiaging agents. Of note, the structural distinction among compounds and combinations of cysteine residues in Keap1 has generated different modifications of cysteine residues. To interpret this mechanism, researchers have proposed the “cysteine code” hypothesis [59], which transforms cysteine modifications into specific biological effects. Deciphering the cysteine code of each Nrf2-activating compound will increase our understanding of its antioxidant function.

Collectively, these findings suggest that Keap1 is an important unit of the Keap1-Nrf2 system that protects cells from oxidative damage through sensing oxidative stress and regulating Nrf2 activity. Studies on the underlying mechanism of Keap1 play a critical role in revealing aging mechanisms and developing antiaging agents.

### 3.2. Competitive Interaction in the Keap1-Nrf2 Axis

Apart from oxidative modifications in the cysteine residues in Keap1, the Keap1 and Nrf2 interaction can also be disrupted via competitive binding. The multifunctional autophagy adapter, sequestosome 1 (p62/SQSTM1), participates in noncanonical activation of Nrf2 [60]. Being involved in the regulation of diverse biological processes, including inflammation, oxidative stress, tumorigenesis, and misfolded protein degradation [61, 62], p62 is a possible initial target of aging interventions, especially because it interacts with the Keap1-Nrf2 pathway. The interaction between Keap1 and p62 was first revealed in 2010 by five independent research groups [63–67]. Like the Keap1-interacting Neh2 domain of Nrf2, p62 contains a KIR motif that allows it to directly interact with Keap1 [63–67]. This competitive interaction leads to sustained accumulation of nonubiquitinated Nrf2 and activation of the antioxidant response [68].

As an important selective substrate for autophagy, p62 is implicated in many models of aging and aging-related diseases [69, 70]. Although p62-promoting autophagy has been linked to a longer lifespan [71–73], direct evidence is very limited. Perhaps, the clearest evidence of its importance in longevity is the premature aging of *p62*^*−*/*−*^ mice [74]. Kwon and colleagues have demonstrated that *p62*^*−*/*−*^ mice exhibit a significantly shortened lifespan and accelerated aging phenotypes. Importantly, the mice also exhibited attenuated NQO1 expression and higher intracellular oxidant levels [74]. These results suggest that p62 may exert its antiaging effects through Keap1-Nrf2 signaling. Recently, one study demonstrated that upregulation of *dp62* (*Drosophila* p62 *homolog ref*(*2*)*P*) from midlife onward significantly increases fly lifespan and reduces mitochondrial ROS levels in flight muscles at 37 days old [75]. However, we do not know whether the decline in ROS levels is related to the competitive interaction between p62 and Keap1. Lamin C (LamC) is one of two nuclear membrane proteins encoded by the *LMNA* gene [76]. Recently, researchers demonstrated that *LamC* mutations lead to a shorter lifespan in *Drosophila* [77]. Furthermore, mutant LamC caused Nrf2 mislocalization and increased p62 levels, generating oxidative stress. In addition, Wei et al. have demonstrated that Keap1/Nrf2/p62 signaling plays an important role in alleviating high inorganic phosphate-induced oxidative stress and subsequent vascular calcification, the latter a complication of aging [78]. Collectively, Keap1/Nrf2/p62 signaling links autophagy to aging, making the pathway a promising antiaging target. Given the complexity of interactions among the Keap1/Nrf2/p62 axis, oxidative stress, and aging, further research is needed to elucidate underlying mechanisms.

In addition to p62, several competing binding proteins of Nrf2 have been reported in recent years. For instance, minichromosome maintenance protein 3 (MCM3) regulates genome replication and redox homeostasis by competing with Nrf2 for Keap1 [79]. Likewise, other Nrf2 competitors can be potential targets for the development of antiaging and anticancer drugs, such as atypical protein kinase C*ι* (aPKC*ι*) [80], inhibitor of apoptosis stimulating protein p53 (iASPP) [81], and family with sequence similarity 129, member B (FAM129B) [82].

Strikingly, both p62 accumulation and Keap1 inhibition mediate Nrf2 activation and participate in aging and aging-related diseases. Additionally, the Keap1 mutation is one of the mechanisms allowing Nrf2 to escape Keap1-mediated repression [83]. These findings indicate that regulation of the Keap1-Nrf2 signaling pathway in higher organisms is a means to promote longevity.

## 4. Nrf2 Phosphorylation

Thus far, the discussed regulatory mechanisms that activate Nrf2 depend on direct interaction with Keap1. However, numerous recent studies have revealed Keap1-independent mechanisms of Nrf2 regulation, including phosphorylation by multiple protein kinases (protein kinase C (PKC), phosphoinositide 3-kinase (PI3K)/protein kinase B (Akt), and glycogen synthase kinase 3*β* (GSK-3*β*)) [84]. Therefore, these protein kinases are part of the aging process. Here, we elaborate and discuss the importance of Nrf2 phosphorylation in aging.

### 4.1. PI3K/Akt/Nrf2

PI3Ks are a family of lipid kinases that play a pivotal role in intracellular signal transduction, controlling many physiological functions and cell processes [85]. The serine/threonine kinase Akt, known as protein kinase B (PKB), is involved in multiple cellular processes, including proliferation, growth, survival, migration, and metabolism [86]. Akt/PKB has emerged as an important signal transduction node in higher eukaryotic cells and is one of the most important and versatile protein kinases at the core of human physiology [87]. Accumulating evidence over the past 20 years suggests that Akt regulates a variety of functions related to longevity and senescence [88, 89]. A downstream target of PI3K/Akt signaling is Nrf2 [90]. Given its well-studied involvement in Nrf2 phosphorylation, the PI3K/Akt/Nrf2 pathway is considered a major way for cells to resist oxidative stress [91]. Thus, the PI3K/Akt/Nrf2 pathway may be a therapeutic target for aging and other conditions related to oxidative stress.

Indeed, PI3K/Akt/Nrf2 signaling plays a central role in aging-related diseases [92, 93]. Many natural compounds exert antioxidant and antiaging effects through the PI3K/Akt/Nrf2 pathway [94, 95]. For instance, naringenin and bungeanum ameliorate behavioral and neurological dysfunction in a D-galactose-induced mouse model of aging through activating this pathway [96, 97]. Through the same pathway, curcumin increases SOD levels and improves premature ovarian failure in mice [98], while anthocyanins potentially target the pathway to ameliorate neurodegenerative diseases [92]. Certain endogenous substances such as thyroid hormone T3 act on the PI3K/Akt/Nrf2 pathway to increase HO-1 [99], whereas erythropoietin and fibroblast growth factors do the same to increase SOD [100, 101]. Because Akt has multiple downstream targets, whether this pathway involves the participation of other signaling molecules remains an open question.

### 4.2. GSK-3*β*

PI3K/Akt signaling regulates Nrf2 activity through GSK-3*β*, a widely distributed serine/threonine kinase encoded by two different genes, alpha (*α*) and beta (*β*) [102, 103]. As a major Akt target, GSK-3*β* is involved in various signaling pathways regulating cell proliferation, apoptosis, glycogen metabolism, and stem cell renewal [104]. Activated Akt phosphorylates and inactivates two GSK-3 subtypes at the *N*-terminus: for GSK-3*α*, position S21 is targeted, whereas GSK-3*β* is phosphorylated at position S9 [105]. GSK-3*β* has many phosphorylation targets and thereby regulates a variety of biological processes involved in several human aging-related diseases, such as cancer, diabetes, and Alzheimer's disease [106]. GSK-3*β* also regulates Nrf2 relocation to the cytosol [107]. When the PI3K/Akt pathway is initiated, Akt first activates GSK-3*β*. The latter then phosphorylates Fyn at threonine residues, leading to Fyn nuclear accumulation [108]. Finally, Fyn phosphorylates Nrf2 at Tyr^568^, allowing Nrf2 to bind with Crm1 and degrade in a Keap1-independent manner [109]. GSK-3*β* also phosphorylates serine residues (334-338) in the Neh6 region of Nrf2 to form a structural motif recognized by SCF/*β*-TrCP E3 ubiquitin ligase, leading to Nrf2 degradation [110].

Increasing evidence demonstrates that GSK-3*β* plays a critical role in aging. GSK-3*α* knockout mice have shorter lifespans than wild-type mice [111]. As a downstream target of GSK-3*β*, Nrf2 is a pivotal molecule in PI3K/Akt/GSK-3*β* signaling [109, 112]. Unlike Akt, GSK-3*β* is active in resting, unstimulated cells [113], and aberrant activation of GSK-3*β* is implicated in aging and neurodegenerative diseases [114, 115]. Inhibition of GSK-3*β* attenuates H_2_O_2_-induced oxidative damage through mediating Nrf2-ARE signaling activation [116, 117]. Xin et al. show that activation of the Akt/GSK-3*β*/Fyn signaling pathway prevents cardiomyopathy through upregulating Nrf2 [108]. Furthermore, adding antisense oligonucleotides targeted to GSK-3*β* improves memory and learning deficits in mice, an effect related to increased nuclear Nrf2, indicating that GSK-3*β* helps maintain healthy aging in rodents [118].

In recent years, the mammalian target of rapamycin complex 2 (mTORC2) has also been found to interact with GSK-3*β* [119]. The mTOR is a highly conserved serine/threonine kinase that responds to changes in energy balance and regulates many cellular functions [120]. There are currently two known mTOR complexes: rapamycin-sensitive mTOR complex 1 (mTORC1) and mTORC2 [121]; the latter is less sensitive to rapamycin and less studied. Among the mTORC2-phosphorylated kinases, such as the protein kinase A/protein kinase G/PKC family, Akt is the most important substrate because of its role in insulin/PI3K signaling [122]. Activation of mTORC2/Akt signaling by 14,15-epoxyeicosatrienoic acid delays endothelial senescence and restores age-dependent endothelial dysfunction [123]. Recently, Yang et al. demonstrated that the mTORC2/Akt/GSK-3*β* pathway is a potential therapeutic target in endothelial senescence [124]. The pathway also plays a major role in cognitive dysfunction [125]. Another protein that inactivates GSK-3*β* and promotes Nrf2 activation is AMP-activated protein kinase (AMPK), involved in cell survival under stress [126]. AMPK also directly phosphorylates Nrf2 at Ser^558^ (Ser^550^ in mice), promoting Nrf2 nuclear accumulation [127].

Collectively, existing research shows that GSK-3*β* is a key node related to aging and aging-related diseases, particularly in terms of the regulatory network centered on the GSK-3*β*/Nrf2 axis. However, the interaction and complexity of multiple signaling pathways currently obscure our understanding of how the GSK-3*β*/Nrf2 axis affects aging. Nevertheless, the characteristics of GSK-3*β* make it an attractive target for the development of antiaging agents.

### 4.3. Other Protein Kinases Involved in Nrf2 Phosphorylation

PKC is a family of serine/threonine kinases that regulates cell proliferation, survival, apoptosis, and migration [128]. Huang and colleagues have found that PKC phosphorylates Nrf2 at Ser^40^ in the Neh2 domain, leading to the dissociation of Nrf2 from Keap1 and consequent Nrf2 nuclear translocation [129]. PKC-*δ* is a novel PKC that is 676-amino acid long [130], has multiple functions in cell signal transduction, and regulates the effects of several different molecules. PKC-*δ*/Nrf2 signaling is a potential therapeutic target because of its antioxidant effect in aging-related diseases, such as osteoarthritis [131] and diabetes [132].

Protein kinase CK2 is involved in a diverse array of biological processes [133], including the cell cycle and cell survival [134, 135]. CK2 is an important regulator of Nrf2 activity [136]. Apopa and colleagues first identified transcription activation domains of Nrf2, Neh4 and Neh5, and then demonstrated that CK2 phosphorylates Nrf2 to trigger the latter's nuclear accumulation [137]. Importantly, downregulation of CK2 activity is closely related to cellular senescence and organismal aging [138, 139], suggesting that the CK2-Nrf2 pathway may be a novel, antiaging target.

Similarly, extracellular signal-regulated kinase (ERK) and mitogen-activated protein kinases, such as p38, both promote Nrf2 activation and upregulate Nrf2 target genes [140, 141]. In particular, p38 phosphorylates Nrf2 at three serine residues (Ser^215^, Ser^408^, and Ser^577^), facilitating a decrease in Nrf2 nuclear accumulation [142]. Modulating this effect through drugs is a potential method for prolonging lifespan [143].

## 5. Crosstalk between mTOR and Nrf2

mTOR, a relatively large (259 kDa) and highly conserved serine/threonine protein kinase regulated mainly by the PI3K/Akt signaling pathway [144], plays a central role in the aging process [121, 122, 145]. Research in the nematode *C*. *elegans* and the fruit fly *Drosophila melanogaster* was the first to show that mTOR regulates lifespan [146, 147]. Nrf2 regulates mTOR through intermediate proteins acting on mTOR posttranslationally [148] and also indirectly upregulates mTOR activity through increasing the expression of the mTOR activator, RagD [149]. However, Bendavit et al. have proposed that Nrf2 directly binds to and thus regulates the mTOR promoter [150]. Furthermore, *β*-TrCP degrades Nrf2 through interaction with the Neh6 region, a structural motif recognized by *β*-TrCP E3 ligase [110]. Qiao and colleagues have shown that mTOR and Nrf2 also interact via the *β*-TrCP/Nrf2 pathway [151], wherein mTOR promotes Nrf2 nuclear translocation through inhibiting *β*-TrCP expression; this process is critical to the initiation and progression of diabetic nephropathy [151]. The Akt/mTORC1/Nrf2 signaling pathway is therefore a valuable therapeutic target for AMD [152, 153], while the Nrf2-miR-129-3p-mTOR axis may be a therapeutic target for autophagy and tumor resistance [154].

Although both mTOR and Nrf2 play an essential role in aging, evidence demonstrating a direct regulatory effect between the two is very limited. Here, we present available studies showing a direct regulatory effect rather than interaction through other signaling pathways. Clarifying the relationship between mTOR and Nrf2 will help elucidate the underlying mechanisms of aging and aging-related diseases.

## 6. *Bach1* and *c*-*Myc*

*Bach1* (BTB and CNC homolog 1) and *c*-*Myc* (a proto-oncogene) are the main negative regulators of Nrf2. During aging, both Nrf2 inhibitors increase [155, 156], suggestive of the mechanism by which age compromises adaptive homeostasis. The ARE motif is recognized by *Bach1*, best known as a repressor of NQO1 expression [157]. Basally, the Bach1/Maf heterodimer competes with the Nrf2/Maf heterodimer for binding to ARE, resulting in the repression or activation of target genes under different cellular conditions [157, 158]. Higher heme levels inhibit *Bach1* DNA-binding activity, leading to *Bach1* dissociation from enhancers and activation of target genes [159]. *Bach1* downregulation protects cells from oxidative stress through enhancing Nrf2/ARE signaling [160]. Thus, *Bach1* plays an important role in the redox induction of HO-1 and NQO1 [157, 161]. Recently, Pomatto and colleagues demonstrated *in vitro* that hyperoxia strongly elevates *Bach1* levels and the competitive effect of *Bach1* and *Nrf2* increases in a time-dependent manner [162]. Additionally, silencing *Bach1* increases basal expression of Nrf2-regulated antioxidant genes in both the young and older human bronchial epithelial cells [163], suggesting that *Bach1* is a potential target for antiaging intervention. In addition to its role in aging, the *Bach1* negative regulation of *Nrf2* is also important in lung cancer metastasis [164, 165].

A transcription factor in the basic helix-loop-helix-leucine zipper (bHLH-LZ) family, *c*-*Myc* regulates the expression of many genes involved in cellular growth and proliferation, as well as DNA damage and genomic instability [166, 167]. Interestingly, *Myc*^*+*/*−*^ mice exhibit an increased lifespan [168]. The *c*-*Myc* is a binding competitor of Nrf2 for ARE sites and regulates Nrf2/ARE signaling through interaction with the ARE-binding complex that increases Nrf2 degradation [169, 170], but until recently, the specific mechanism of *c*-*Myc* effects on aging was unknown.

Collectively, as known inhibitors of Nrf2-regulated transcription, increases in Bach1 and c-Myc reflect declining Nrf2 effectiveness in adaptive homeostasis during aging. Davies and Forman have proposed that although *Bach1* and *c*-*Myc* expression increased with age, continual adaptive homeostasis contributes to lower cancer risk [171]. Additionally, Pomatto et al. believe that changes in the balance of *Nrf2*, *Bach1*, and *c*-*Myc* levels may be the cause of serious disturbances to the stress response and adaptive homeostasis during chronic hyperoxia and aging [162]. Nevertheless, research has mainly focused on quantitative changes when examining the interaction between the two inhibitors, Nrf2, and aging. We require further exploration on how increased *Bach1* and *c*-*Myc* expression generates aging-related changes through Nrf2 signaling.

## 7. MicroRNAs (miRNAs) in the Regulation of Keap1-Nrf2 Signaling

The miRNAs are small noncoding RNA sequences containing approximately 22-24 nucleotides. They inhibit gene expression by binding to complementary sequences in the 3′-untranslated region (UTR) of target mRNA and modulate many biological functions [172]. A growing body of evidence shows that miRNAs play key roles in and may thus be drug discovery targets for aging and age-related diseases. miR-34a, miR-100, and miR-21 are involved in endothelial senescence and upregulated in senescent cells [173, 174]. Through downregulation of GSK-3*β* and upregulation of Nrf2 signaling, miR-135a and miR-135b-5p, respectively, alleviate neuronal damage from oxygen-glucose deprivation and reoxygenation [175, 176]. Additionally, high expression of miR-200a inhibits Keap1 and activates the Nrf2 antioxidant pathway, thereby preventing lipids from accumulating oxidative damage [177]. Moreover, miR-941 is a novel Keap1-targeting miRNA that protects cells from oxidative stress through inhibition of Keap1 3′-UTR expression, thus activating the Nrf2 cascade [178]. miR-144 is a therapeutic option for retinal degenerative diseases [179]. Numerous miRNAs participate in the regulation of the Keap1-Nrf2 pathway for oxidative stress. Additional studies will likely uncover more miRNAs that are involved in regulating Nrf2/ARE signaling through targeting Nrf2, Keap1, or associated proteins [180]. Currently, less information is available on aging-dependent miRNA changes that target Nrf2 or Keap1 signaling. Interactions involving miRNA are complex because each miRNA has a variety of target genes, while being controlled by multiple upstream signals. Moreover, miRNA expression can differ across tissues or species. Despite the potential difficulties in untangling these networks, miRNAs are clearly an attractive field for studies on aging-related disease therapy.

## 8. Antioxidant Ingredients in the Diet

Diet is an important regulatory factor during the aging process [181]. Dietary calorie restriction and dietary antioxidants are advantageous for healthy aging and longevity by decreasing oxidative stress and modulating age-related signaling pathways [182]. Dietary polyphenols (phenolics), the general term for a variety of chemical substances found in plants, have been considered potential antiaging compounds due to their prominent antioxidant capacity [182, 183]. For example, curcumin, a polyphenolic compound isolated from *Curcuma longa*, has been shown to exert antiaging characteristics [184]. Research has shown that curcumin is a hormetic agent that stabilizes Nrf2 and enhances the expression of HO-1 [185, 186]. Furthermore, curcumin upregulates the antiapoptotic Bcl-2 protein and downregulates the proapoptotic proteins, Bax and caspase-3 [187]. The oxidative stress biomarkers, including MDA (malondialdehyde), SOD, and GSH, are upregulated (SOD, GSH) or downregulated (MDA) by curcumin [188]. The antioxidant activities of catechins have also been extensively studied [189]. Of these, (−)-epigallocatechin-3-gallate (EGCG), the principal catechin in green tea, has received the most attention. Sun and colleagues demonstrated that EGCG can activate Nrf2 by binding to Keap1, which plays a key role in preventing diabetic nephropathy [190]. Additionally, EGCG can activate other factors, such as Akt and ERK, which may also result in Nrf2 activation [191]. Resveratrol is the most widely studied polyphenol and is considered an important compound for life extension and anticancer and cardioprotection treatments. These three properties are inseparable from its ability to reduce oxidative stress [192]. Resveratrol can stimulate the activity of Nrf2, upregulate a variety of antioxidant enzymes such as NQO1 and HO-1, and control *γ*-glutamylcysteine synthetase (GCLC), the enzyme that regulates glutathione synthesis [193]. On the one hand, resveratrol can regulate Nrf2 by activating the PI3K/Akt signaling pathway to increase Nrf2 stabilization, and on the other hand, it can also upregulate the expression of p62, which can compete with Nrf2 to bind to the Keap1-Kelch domain, thereby promoting Nrf2 nuclear translocation [194]. Other bioactive polyphenols including tannic acid, wogonin, ampelopsin, (-)epicatechin, ellagic acids, lignans, rosmarinic acid, and their derivatives are also potentially important for healthy human aging and longevity.

Dietary flavonoids, also major antioxidant phytochemicals, are closely associated with antiaging due to their direct antioxidant capacity [195]. More than 8000 naturally occurring flavonoids have been identified from various vegetables, fruits, and plants [196]. Many natural flavonoids, including flavones, flavonols, chalcones, and isoflavones, have been identified to be Nrf2 activators and are thus regarded to be potential antiaging agents [195, 197]. For instance, quercetin upregulates the expression of Nrf2, HO-1, NQO1, and GCLC to resist oxidative stress. Quercetin could restore the serum and tissue activities of SOD, GSH-Px, and CAT and reduce ROS and MDA levels to different extents in Nrf2 wild-type model mice of dry, age-related macular degeneration [198]. Moreover, a recent study showed that quercetin and catechin can not only directly but also indirectly regulate the expression of Nrf2, HO-1, and NQO1 via two miRNA molecules, miR-25-3p and let-7a-5p [199]. Similarly, other widely investigated flavonoids, such as apigenin [200], baicalin [201], kaempferol [202], genistein [203], dihydroquercetin [204], and procyanidins [205], protect cells and tissues against oxidative injury via activation of the Nrf2 signaling pathway. Notably, baicalin and baicalein can increase Nrf2 protein levels by upregulating the expression of p62 protein and phosphorylating ERK1/2 and PKC [206].

Some triterpenoids also have obvious antioxidant and antiaging activity. For instance, Xu et al. demonstrated that ganoderic acid D exhibits potent antisenescence effects against H_2_O_2_-induced premature senescence of human amniotic mesenchymal stem cells through the activation of PERK/Nrf2 signals [141]. Additionally, Lefaki and colleagues demonstrated that 18*α*-glycyrrhetinic acid can activate the Nrf2/SKN-1 pathway against DNA damage [207]. These findings suggest that dietary triterpenoids could be used in a prophylactic or therapeutic strategy against aging or aging-related diseases.

Dietary constituents activate the Nrf2/ARE pathway by a variety of mechanisms, but most of the activators act by stimulating the phosphorylation of Nrf2, leading to the dissociation of Nrf2 from Keap1. For example, curcumin and resveratrol can phosphorylate Nrf2 through the PI3K/Akt signaling pathway. Phosphorylated Nrf2 translocates into the nucleus and binds to ARE/sMaf, promoting the transcription of ARE-driven genes and thereby alleviating oxidative stress-mediated damage [187, 191, 194]. Similarly, dietary components can also activate AMPK, CK2, PKC, ERK, and p38 signaling pathways to cause Nrf2 phosphorylation and nuclear translocation, thus promoting the transcription of antioxidant genes [141, 204, 206, 208]. Additionally, certain dietary phytochemicals such as resveratrol can react with the cysteine residues of Keap1 (i.e., Cys151, Cys257, Cys273, Cys288, and Cys297) via oxidation or alkylation to dissociate Nrf2 from Keap1 [208, 209]. Flavonoids baicalin and baicalein can increase Nrf2 protein levels by upregulating the expression of p62 protein [206], which contains a KIR motif that allows it to compete with Nrf2 to bind to Keap1 and obstruct the formation of Nrf2-Keap1 complex [210]. Other dietary constituents such as EGCG promote Nrf2 into the nucleus by competing with Keap1 [190]. Therefore, exploring the effects of dietary components on the activation of the Nrf2/ARE pathway is crucial to elucidate the mechanisms of these dietary antioxidants in exerting chemopreventive effects.

Taken together, there is increasing evidence that dietary antioxidant phytochemicals exert positive effects against age and age-related diseases. The intracellular Keap1/Nrf2/ARE signaling pathway plays a vital role in this activation process. Generally, these dietary antioxidant phytochemicals exert their antiaging effects by targeting Nrf2 signals. However, most studies mainly focus on *in vitro* experimental models. So far, only a few *in vivo* studies have been conducted on the antiaging effects. Moreover, the activation of the Nrf2/ARE pathway is not always beneficial because certain flavonoids may also promote the growth of cancer cells [208]. Therefore, the relationship between the influence of dietary antioxidant intake on the Nrf2/ARE pathway and the physiological effect still remains unclear. In addition, some dietary antioxidants such as flavonoids undergo sulfation, glucuronic acid oxidation, and methylation in the intestinal cells of the small intestine and liver, thereby producing different metabolites [211]. Therefore, this stage of metabolism changes the bioavailability of the parent compound. Elucidating these mechanisms will require more *in vivo* studies.

## 9. Conclusions

**Figure 3:**
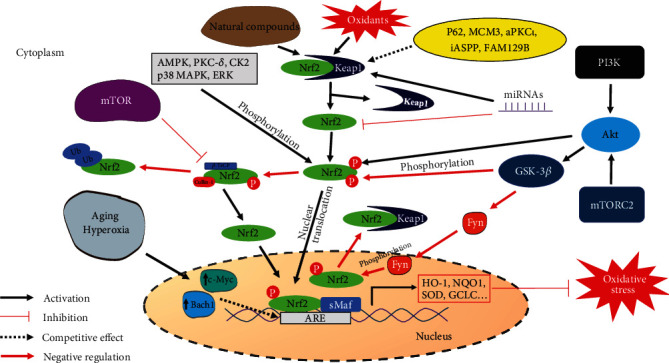
The crosstalk between Keap1/Nrf2 and other signaling pathways involved in oxidative stress and aging.

Elevated oxidative stress in older animals is considered a hallmark of aging. The Keap1-Nrf2 signaling pathway is the most important cellular antioxidant system, controlling the expression of various antioxidant enzymes. Therefore, Keap1-Nrf2 signaling is a potential pharmacological target for the development of antiaging therapies. Keap1 regulation, especially via its component cysteines, is mainly restricted to the cytoplasm. Cracking the “cysteine code” in Keap1 is a major undertaking that is expected to clarify underlying mechanisms of action of certain antiaging agents. In contrast, Nrf2 regulation is not limited to the cytoplasm, and nuclear regulation through *Bach1* and *c*-*Myc* is also very important. Furthermore, various factors regulate Nrf2 signal transduction, including Nrf2 protein stability, phosphorylation, nuclear export, and Nrf2-ARE complex formation (Figure 3). The Keap1-Nrf2 signaling pathway may also closely interact with other signaling pathways, such as PI3K/Akt/GSK-3*β* and ERK. The direct interaction between Nrf2 and mTOR may be an attractive area of study to uncover antiaging therapeutic targets.

Although scientists have achieved unprecedented results in elucidating the Keap1-Nrf2 signaling pathway, many key issues remain unresolved. For example, is there a more efficient domain in Keap1 that regulates its conformational changes? Are there other specific molecules involved upstream or downstream of Nrf2? Why does Nrf2 decrease with age? Moreover, we know next to nothing about the possible effects of miRNAs on the Nrf2 pathway in elderly individuals. Additionally, the relationship between the influence of dietary compounds on the Keap1/Nrf2/ARE signaling pathway and its physiological effects and how to improve their bioavailability still needs more research. Understanding these mechanisms should provide new tools for developing interventions that improve the quality of life in older adults.
